# *Actaea racemosa* L. Rhizome Protect against MPTP-Induced Neurotoxicity in Mice by Modulating Oxidative Stress and Neuroinflammation

**DOI:** 10.3390/antiox12010040

**Published:** 2022-12-25

**Authors:** Marika Cordaro, Ramona D’Amico, Roberta Fusco, Tiziana Genovese, Alessio Filippo Peritore, Enrico Gugliandolo, Rosalia Crupi, Davide Di Paola, Livia Interdonato, Daniela Impellizzeri, Salvatore Cuzzocrea, Rosanna Di Paola, Rosalba Siracusa

**Affiliations:** 1 Department of Biomedical, Dental and Morphological and Functional Imaging, University of Messina, 98125 Messina, Italy; 2 Department of Chemical, Biological, Pharmaceutical and Environmental Sciences, University of Messina, 98166 Messina, Italy; 3 Department of Veterinary Science, University of Messina, 98168 Messina, Italy; 4 Department of Pharmacological and Physiological Science, Saint Louis University School of Medicine, Saint Louis, MO 63104, USA

**Keywords:** redox balance, neuroinflammation, neurodegeneration, dietary supplement

## Abstract

Parkinson’s disease (PD) is a dopaminergic neuron-related neurodegenerative illness. Treatments exist that alleviate symptoms but have a variety of negative effects. Recent research has revealed that oxidative stress, along with neuroinflammation, is a major factor in the course of this disease. Therefore, the aim of our study was to observe for the first time the effects of a natural compound such as *Actaea racemosa* L. rhizome in an in vivo model of PD induced by neurotoxin 1-methyl-4-phenyl-1,2,3,6-tetrahydropyridine (MPTP). For the study, mice received four injections of MPTP (20 mg/kg) for the induction of PD. Starting 24 h after the first administration of MPTP we treated mice with *Actaea racemosa* L. rhizome (100 mg/kg) daily for seven days. Our findings clearly demonstrated that *Actaea racemosa* L. rhizome treatment decreases oxidative stress by activating redox balance enzymes such as Nrf2/HO-1. We also demonstrated that *Actaea racemosa* L. rhizome is capable of modulating inflammatory indicators involved in PD, such as IκB-α, NF-κB, GFAP and Iba1, thus reducing the degeneration of dopaminergic neurons and motor and non-motor alterations. To summarize, *Actaea racemosa* L. rhizome, which is subject to fewer regulations than traditional medications, could be used as a dietary supplement to improve patients’ brain health and could be a promising nutraceutical choice to slow the course and symptoms of PD.

## 1. Introduction

Parkinson’s disease (PD), which primarily affects the substantia nigra (SN) of the brain, is one of the most common neurodegenerative diseases [1]. This area is important for producing dopamine which controls motor skills. PD occurs in patients over the age of 50 with symptoms such as tremor at rest, slowed mobility and loss of balance [2]. The etiology of PD remains largely unknown. Brain injury and exposure to various environmental factors such as pesticides and neurotoxins are potential causes [3,4]. Increased oxidative stress and inflammatory processes have been linked to PD pathology in general [5]. Current treatments for PD, including the best-known, levodopa, carbinoxamine, cicrimin and carbidopa, are capable of slowing the progression of the disease and managing symptoms but have numerous side effects such as stress, apprehension and lethargy, which have a negative impact on the patient health [6,7]. Studies are therefore needed to evaluate new compounds capable of acting both on neuroinflammation and on the redox imbalance responsible for neurodegeneration in patients with PD. Several research findings have recently reported on how medicinal plant-based therapies have beneficial effects on various pathologies, including neurodegenerative ones [8,9,10,11,12,13]. Natural substances have been shown to alleviate tremors and improve cognitive performance. Plants such as *Mucuna pruriens*, *Hyoscyamus niger*, *Sida cordifolia* and *Withania somnifera* have been used to treat PD [14,15]. Metal chelation and antioxidant activities have been demonstrated in *Mucuna pruriens* seed extract [16]. *Hyoscyamus niger* seed extract contains active compounds such as hyoscine and hyoscyamine and has an anticholinergic effect [17]. The root extracts of *Sida cordifolia* and *Withania somnifera* also showed antioxidant effects [18,19,20]. Other plants with a high antioxidant power, such as *Gingko biloba*, green tea, and berries have also been used for the treatment and slowing of the progression of PD [21,22].

The rhizomes and dried roots of *Actaea racemosa* L. (the synonym *Cimicifuga racemosa* (L.)) are a well-studied pharmaceutical herbal drug. They are also extensively used as a food supplement. The plant is native to the eastern United States and was historically used by Native American women to relieve pain during menstruation or childbirth. This plant has also been used for the treatment of kidney disorders, rheumatism and malaria, but also general malaise [23,24]. At the beginning of the twentieth century, *Actaea racemosa* L. was also introduced as a phytomedicine in Europe to counteract the symptoms related to premenstrual syndrome, dysmenorrhea, and menopause [25,26,27,28]. A recent clinical study also supports the safety and efficacy of *Actaea racemosa* L. [29,30]. Pharmacological studies on *Actaea racemosa* L. show that the plant’s biological effects are caused by substances with dopaminergic [31] and serotonergic [32,33] activity rather than an estrogen-like activity. Several techniques have been used for the characterization of *Actaea racemosa* L. [34]. The phytochemicals present in the rhizomes of *Actaea racemosa* L. have been well studied in recent years despite the many difficulties in characterizing the various constituents due to the lack of specific standards [34,35,36]. However, these studies have revealed that the main elements of *Actaea racemosa* L. rhizomes are triterpene glycosides, phenols, flavonoids, and alkaloids, among others. As minor compounds, *Actaea racemosa* L. contains aromatic acid (ferulic acid, iso ferulic acid, caffeic acid, and methyl esters of caffeic acid), cinnamic acid esters (cimicifugic acid, cimicifugic acid A–F, cimiracemates A–D, fukiic acid, piscidic acid, and fukinolic acid), resin, phytosterol, fatty acid, starch, and sugar [37,38]. Furthermore, *Actaea racemosa* L. also contains quinolinic and quinolizidine alkaloids, anagyrine, baptifolin, magnoflorine, methylcystine, methyl serotonin, and other alkaloids.

Triterpenes and saponins have been shown to possess a range of biological actions such as anti-inflammatory and anticancer effects [39,40]. Among the most abundant triterpenes found in the root and in the rhizome of *Actaea racemosa* L. are actein and 23-epi-26-deoxyactein, which seem to be the main ones responsible for its biological action [41,42,43,44,45]. Most of the research focuses only on triterpenes as an active ingredient in the *Actaea racemosa* L. extract, which are believed to be responsible for pharmacological action. However, other constituents present in *Actaea racemosa* L. can be explored for their pharmacological activity. For example, alkaloids are important constituents of natural products that have established biological action, mainly as CNS agents. There are more than one hundred nitrogen compounds in the *Actaea racemosa* L. extract [46]. The presence of guanidine-type alkaloids in the extract is one of the distinctive features of the *Actaea racemosa* L. metabolome. Even the phenolic acids present in the extract may possibly act as counter-ions of positively charged alkaloids and create strong pairs of ions which are responsible for the biological activity of the extract.

In recent years, *Actaea racemosa* L. has also been shown to have a high antioxidant power due to its activity of metal chelation and radical scavenging demonstrated in in vitro experiments [47]. Studies with *Actaea racemosa* show a significant decrease in neuronal damage by several mechanisms such as increased superoxide dismutase and catalase activities, suppression of nuclear factor kappa B (NF-κB), interleukin 1 (IL-1), glial fibrillary acid protein (GFAP) and IL-6 expression in a rat model of traumatic brain injury [48]. Furthermore, in both in vitro and in vivo study it was demonstrated that a compound containing 10 herbs including *Cimicifuga heracleifolia*, called Yeoldahanso-tang, can be used when modified for both prevention and treatment of neurodegenerative diseases with PD as it is in capable of enhancing autophagy and having a neuroprotective effect [49].

Therefore, based on the characteristics of *Actaea racemosa* L. reported in the literature and of the studies that have highlighted the neuroprotective effect of natural compounds, and in particular of *Cimicifuga*, we hypothesized that the rhizome of this extract compound could effectively suppress the oxidative stress and neuroinflammation typical of PD.

## 2. Material and Methods

### 2.1. Animals

C57/BL6 mice (male 25–30 g; Envigo, Milan, Italy) were acclimated in a controlled setting using conventional rodent food and water. Mice were housed in steel cages in a room set at 22 ± 1 °C with 12 h day and night cycles. The study was approved by the University of Messina’s Animal Care Review Board (OPBA). All in vivo tests were conducted in accordance with the new instructions of the United States, Europe, Italy, and the ARRIVE guidelines (approval code: n° 89/2021-PR).

### 2.2. MPTP-Induced PD and Treatment

MPTP or saline were given to mice as previously described [50]. For treatment we used *Actaea racemosa* L. rhizome extract (ARL rhizome) at the dose of 100 mg/kg (origin of raw material USA; the rhizome is harvested and dried; company Fontana, Canosa di Puglia, Italy, Batch Number: 82114885). Mice were given this compound orally, beginning 24 h after the 1st MPTP injection and continuing for 7 days after the last MPTP administration. For MPTP intoxication, mice received four intraperitoneal (i.p.) injections of MPTP (20 mg/kg; Sigma, St. Louis, MO, USA) in saline at 2 h intervals over the course of one day: total dose per mouse was 80 mg/kg. Only the vehicle was given to the control animals. Mice were euthanized 8 days following MPTP injection, and brains were taken and processed for biochemical and molecular examination. The MPTP (20 mg/kg) and ARL rhizome (100 mg/kg) doses used were based on research in the literature and on preliminary studies conducted in our laboratories [51,52].

#### Experimental Groups

The animals were randomly distributed into the following groups:

Group 1. *Sham + Veh*: vehicle solution (saline) was injected i.p. during the 1st day, as in MPTP protocol.

Group 2. *Sham +* ARL rhizome: identical to Group 1, but ARL rhizome extract (100 mg/kg body weight, orally) was given starting 24 h after the first saline solution injection and continuing for 7 days after the last administration of vehicle solution (data are not shown in all results because of no significant differences with Group 1).

Group 3. *MPTP + Veh*: MPTP was administered as described for Group 1.

Group 4. *MPTP+* ARL rhizome: same as Group 3, but *Actaea racemosa* L. rhizome extract (at a dose of 100 mg/kg, orally) was administered, starting 24 h after the 1st MPTP administration and continuing through 7 additional days after the last dose of MPTP.

### 2.3. Western Blot Analysis

Western blot analysis was carried out exactly as previously described [53,54]. Primary antibodies used were: anti-nuclear factor erythroid 2–related factor 2 (Nrf2) (1:500; Santa Cruz Biotechnology, Heidelberg, Germany), anti-NAD(P)H Quinone Dehydrogenase 1 (NQO1) (1:500; Santa Cruz Biotechnology, Heidelberg, Germany), anti-heme oxygenase-1 (HO-1) (1:500; Santa Cruz Biotechnology, Heidelberg, Germany), anti-Ikb-α (1:500; Santa Cruz Biotechnology, Heidelberg, Germany), antinuclear factor-κB (NF-κB) p65 (1:1000; Santa Cruz Biotechnology, Heidelberg, Germany), GFAP (1:1000; Santa Cruz Biotechnology, Heidelberg, Germany), anti-ionized calcium binding adaptor molecule 1 (Iba1) (1:1000; Santa Cruz Biotechnology, Heidelberg, Germany), anti-Bax (1:500; Santa Cruz Biotechnology, Heidelberg, Germany), anti-Bcl-2 (1:1000; Santa Cruz Biotechnology, Heidelberg, Germany). The membranes were then incubated with IgG peroxidase-conjugated secondary antibody-conjugated bovine mouse IgG or IgG peroxidase-conjugated goat anti-rabbit (1:2000, Jackson ImmunoResearch, Cambridge, UK). Probing the membranes with a b-actin or laminin antibody revealed the protein content. The manufacturer’s advanced chemiluminescence detection system reagent was used to detect the target antibody signals (SuperSignal West Pico Chemiluminescent Substrate, Pierce, Waltham, MA, USA). The expression of the protein bands was determined by densitometry and adjusted to b-actin and laminin levels using BIORAD ChemiDocTM XRS Plus software (Image Quant TL, v2003, Segrate, Italy). Blot signal images (8-bit resolution/600 dpi) were imported into the analysis application [55,56].

### 2.4. Immunohistochemical Analysis for the Localization of GFAP, Iba1, Tyrosine Hydroxylase (TH), Dopamine Transporter (DAT), α-Synuclein (α-Syn)

The immunohistochemical study was performed as previously described [57,58]. Primary antibodies used were: anti-GFAP (Santa Cruz Biotechnology, 1:100 in PBS, *v/v*), anti-Iba1 (Santa Cruz Biotechnology, 1:100 in PBS, *v/v*), anti-TH (Millipore, 1:500 in PBS, *v/v*, Burlington, MA, USA), anti-DAT (Santa Cruz Biotechnology, 1:300 in PBS, *v/v*), anti-α-syn (Santa Cruz Biotechnology, 1:100 in PBS, *v/v*). The slices were then rinsed with PBS and treated with secondary antibody the next day. A biotin-conjugated goat anti-rabbit IgG and an avidin-biotin peroxidase complex were used to identify specific labeling (Vector). Five stained sections from each mouse were scored blindly and examined using a Leica DM6 microscope (Leica Microsystems SpA, Milan, Italy) in accordance with standard procedures [59,60]. The histogram profile is related to the positive pixel intensity value obtained.

### 2.5. Immunofluorescence Co-Localization for TH/α-Syn and DNA Fragmentation (TUNEL) Assay for Dopaminergic Neurons

The immunofluorescence analysis was carried out according to the methodology published previously [61]. Anti-TH antibody (1:100; Invitrogen, 185, Waltham, MA, USA) and anti-α-syn (1:100; Invitrogen, PA5-85791) were incubated in a humidified chamber at 37 °C O/N on the brain sections. After washing with PBS, sections were incubated for 1 h at 37 °C with secondary antibodies, TEXAS RED-conjugated anti-rabbit Alexa Fluor-594 (1:1000 in PBS, *v/v* Molecular Probes, Eugene, OR, USA) and FITC-conjugated anti-mouse Alexa Fluor-488 (1:2000 *v/v* Molecular Probes, Eugene, OR, USA). Nuclei were stained by adding 2 μg/mL 40,60-diamidino-2-phenylindole (DAPI; Hoechst, Frankfurt, Germany) in PBS. To identify dopaminergic neurons subjected to DNA fragmentation, some sections were sequentially doubled by immunostaining with anti-TH followed by TUNEL. Sections were equilibrated in labeling buffer for 10 min at room temperature immediately after the last immunostaining rinse, and then incubated in the TUNEL staining solution according to a Roche technique [62,63]. Sections were washed with PBS and were incubated with secondary antibody FITC-conjugated anti-mouse Alexa Fluor-488 antibody (1:2000 *v/v* Molecular Probes, Eugene, OR, USA) for 1 h at 37 °C. The sections were analyzed using a fluorescence microscope (Leica DM2000, Wetzlar, Germany). Each photograph was scanned with an 8-bit resolution in a 2560 × 1920 pixel array. The collected photos were then cut out and processed for figure assembly using Adobe Photoshop 7.0. (Adobe Systems, Palo Alto, CA, USA) [64].

### 2.6. Histological Analysis

Eight days after MPTP injection, brain tissue was collected. As previously described, the sections were stained with Hematoxylin/Eosin (H&E) and studied using light microscopy interfaced to an imaging system [65,66]. Blinded observation was used for histological assessment, and slides were graded for the severity of pathological outlines following H&E staining using a semiquantitative 5-point scale: 0 = no pathology; 1 = mild pathology; 2 = moderate pathology; 3 = severe pathology; 4 = extremely severe pathology.

### 2.7. Behavioral Tests

Behavioral studies on mice were conducted one day before MPTP injection and eight days following PD induction. All behavioral studies were conducted in a blinded manner.

#### 2.7.1. Analysis for Motor Symptoms

- Open Field (OF)

Using a previously published technique [67,68], locomotor activity was observed in an OF for 5 min. The animals’ actions were recorded. The number of line crossings and the amount of time spent in the center were calculated and scored.

- Rotarod Test (RT)

Motor incoordination was assessed with a rotary rod apparatus using a protocol previously described [50,69]. Each mouse’s total time on the RT and total number of falls were recorded.

- Catalepsy Test (CT)

Catalepsy, defined as a decreased ability to initiate movement and a failure to maintain proper posture, was measured as previously described [70,71]. The time the mice remained in this position was timed using a timer up to 180 s. The duration the mice stayed in this position was timed up to 180 s. Cataleptic mice were those who remained in this position for 30 s or longer.

- Pole test (PT)

The PT was performed as previously described [53,72]. Two criteria were evaluated: time until the mouse turned by 180°, and time until the mouse descended to the floor.

#### 2.7.2. Analysis for Non-Motor Symptoms

- Elevated plus maze (EPM)

As previously mentioned, the EPM test was used to assess anxiety-like behavior [73,74]. A researcher who was not aware of the animal treatment scored the proportion of time spent on the open arms. Anxiety was indicated by a reduction in the proportion of time spent in the open arms as well as a reduction in the proportion of admissions into the open arms.

- Marble burying test (MBT)

The marble burying test is a defensive behavioral test for mice used to determine anxiety levels. The analysis was carried out exactly as detailed in a prior paper [75]. For 30 min, the mice were placed in a cage with a 5 cm thick coating of pine and 20 little glass marbles (15 mm in diameter) set in 4 evenly spaced rows of 5 marbles each. A significant number of marbles buried suggests increased concern. As a result, an animal is considered worried if at least 2/3rd of the marble is covered in litter.

- Tail suspension test (TST)

We used the TST for additional neuropsychological tests, such as depression. This behavioral test was carried out exactly as previously reported [76]. Except for breathing movements, all body motions were recorded for 6 min.

### 2.8. Statistical Analysis

All values in the figures and text are expressed as mean ± standard deviation (SD) of N observations. For the in vivo studies, N represents the number of animals studied. In experiments involving histology, immunohistochemistry, and immunofluorescence the figures shown are representative of at least three experiments performed on different days on tissue sections collected from all animals in each group. The results were analyzed by one-way ANOVA followed by a Bonferroni post hoc test for multiple comparisons. A *p*-value of less than 0.05 was considered significant [77].

## 3. Results

### 3.1. Effects of Actaea racemosa L. Rhizome on Nrf2/HO-1/NQO1 Pathway

**Figure 1:**
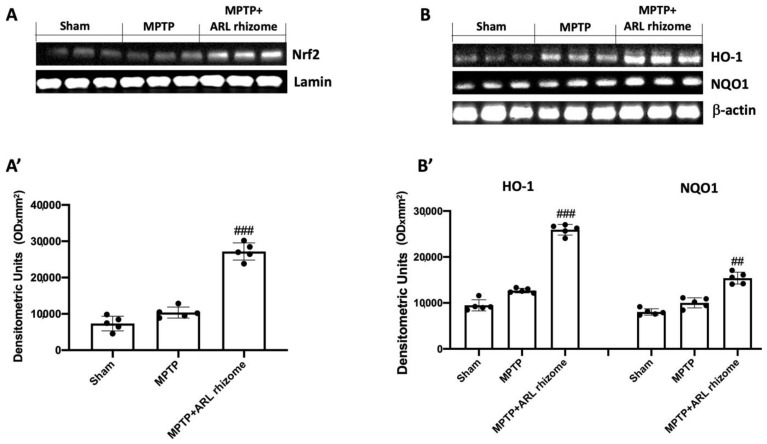
The impact of ARL rhizome on Nrf2/HO-1/NQO1 pathway expression after MPTP-injection. Representative Western blots were carried out for nuclear Nrf2 (**A**,**A’**), cytoplasmic HO-1, and NQO1 (**B**,**B’**) expression. A demonstrative blot of lysates (five animals/group) is given, along with a densitometric analysis for all animals (**A’**,**B’**). Values = means ± SD of five animals in each group. ## *p* < 0.01 vs. MPTP; ### *p* < 0.001 vs. MPTP.

In general, oxidative stress in the brain is associated with increased expression of genes involved in free radical detoxification as well as genes responding to cell survival stress. In this regard, we wanted to see how ARL rhizome treatment affected Nrf2 activation and, as a result, the expression of HO-1 and NQO1. When MPTP-treated mice were compared to Sham mice, Western blot analysis demonstrated a slight increase in Nrf2. This demonstrates that the animal’s body attempts to activate an antioxidant response following treatment with MPTP even if this is not significant. Treatment with ARL rhizome was able to enhance the antioxidant response by significantly increasing Nrf2 levels (Figure 1A and densitometric analysis Figure 1A’). Furthermore, the same results were observed for HO-1 and NQO1. Thus, a nonsignificant increase was found in the MPTP group, while in mice treated with ARL rhizome the levels of these proteins were significantly increased (Figure 1B, and densitometric analysis Figure 1B’).

### 3.2. Effect of Actaea racemosa L. Rhizome Treatment on Neuroinflammation

**Figure 2:**
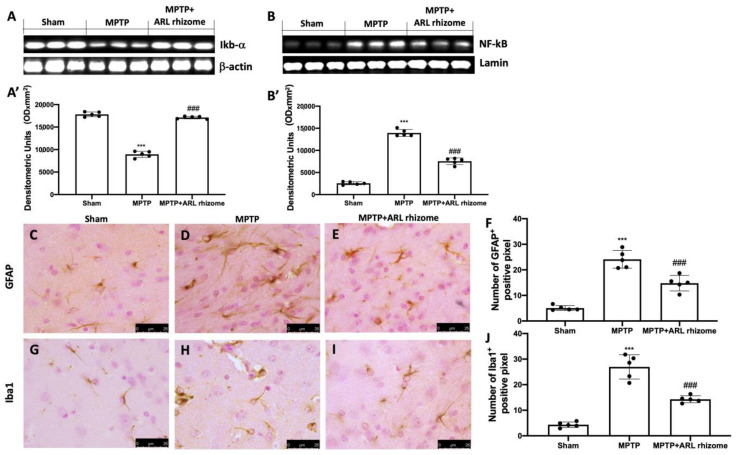
The effects of ARL rhizome on NF-kB pathway and the activation of astrocytes and microglia following MPTP injection. Western blots were used to look for cytoplasmic Ikb-a (**A**,**A’**) and nuclear NF-kB (**B**,**B’**) expression. A demonstrative blot of lysates (five animals/group) is given, along with a densitometric analysis for all animals (**A’**,**B’**). GFAP immunohistochemistry was performed in Sham (**C**), MPTP (**D**), and MPTP + ARL rhizome (**E**). The findings are shown as the number of GFAP + positive pixels (**F**). The same analysis was carried out on Iba-1 brain slices from the Sham (**G**), MPTP (**H**), and MPTP + ARL rhizome (**I**) groups. The results are given in terms of the number of Iba-1+ positive pixels (**J**). The images are figurative of at least three independent experiments. Values = means ± SD of 5 animals in each group. *** *p* < 0.001 vs. Sham; ### *p* < 0.001 vs. MPTP.

We used Western blot analysis on midbrain tissues to assess the anti-inflammatory effect of ARL rhizome treatment on the development of PD. When MPTP-injured animals were compared to Sham mice, Ikb-a degradation was triggered, whereas ARL rhizome treatment elevated Ikb-a cytosolic expression (Figure 2A, and densitometric analysis Figure 2A’). As a result of increased Ikb-a degradation, we saw a considerable increase in nuclear translocation of NF-kB in the MPTP-treated group, but ARL rhizome treatment reduced its expression in the nucleus (Figure 2B, and densitometric analysis Figure 2B’). In addition, immunohistochemistry labeling for GFAP and Iba-1 expression was used to examine astrocyte and microglial cell activity in connection to PD. The levels of GFAP and Iba-1 were very low in the Sham group but significantly higher in the MPTP-treated mice. ARL rhizome treatment effectively reduced the elevated expression of GFAP and Iba-1 under these conditions (Figure 2C–F for GFAP and Figure 2G–J for Iba-1).

### 3.3. Effect of Actaea racemosa L. Rhizome Treatment on Apoptosis

**Figure 3:**
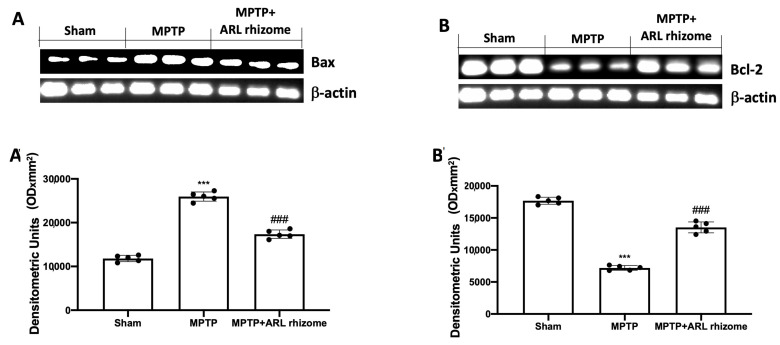
Effect of ARL rhizome on Bax and Bcl-2 expression after MPTP-injection. Western blots for Bax (**A**,**A’**) and Bcl-2 (**B**,**B’**) expression were performed. A demonstrative blot of lysates (five animals/group), with a densitometric analysis for all animals, is shown (**A’**,**B’**). Values = means ± SD of five animals in each group. *** *p* < 0.001 vs. Sham; ### *p* < 0.001 vs. MPTP.

To assess the effect of ARL rhizome treatment on MPTP-induced apoptosis, we used Western blot analysis to look at the expression of Bax and Bcl-2. Tissues from MPTP-treated animals had higher levels of Bax expression than the Sham group. This expression was diminished by ARL rhizome treatment (Figure 3A and densitometric analysis Figure 3A’). Tissues from Sham mice, on the other hand, displayed basal levels of Bcl-2; MPTP injection reduced this expression, while ARL rhizome treatment restored Bcl-2 expression to baseline (Figure 3B, and densitometric analysis Figure 3B’).

**Figure 4:**
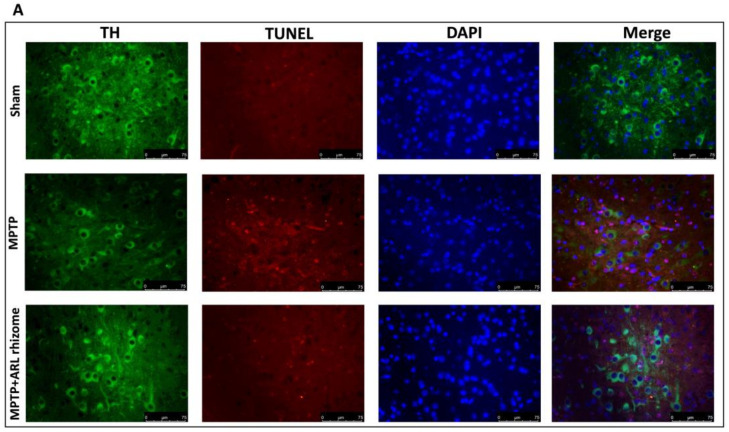

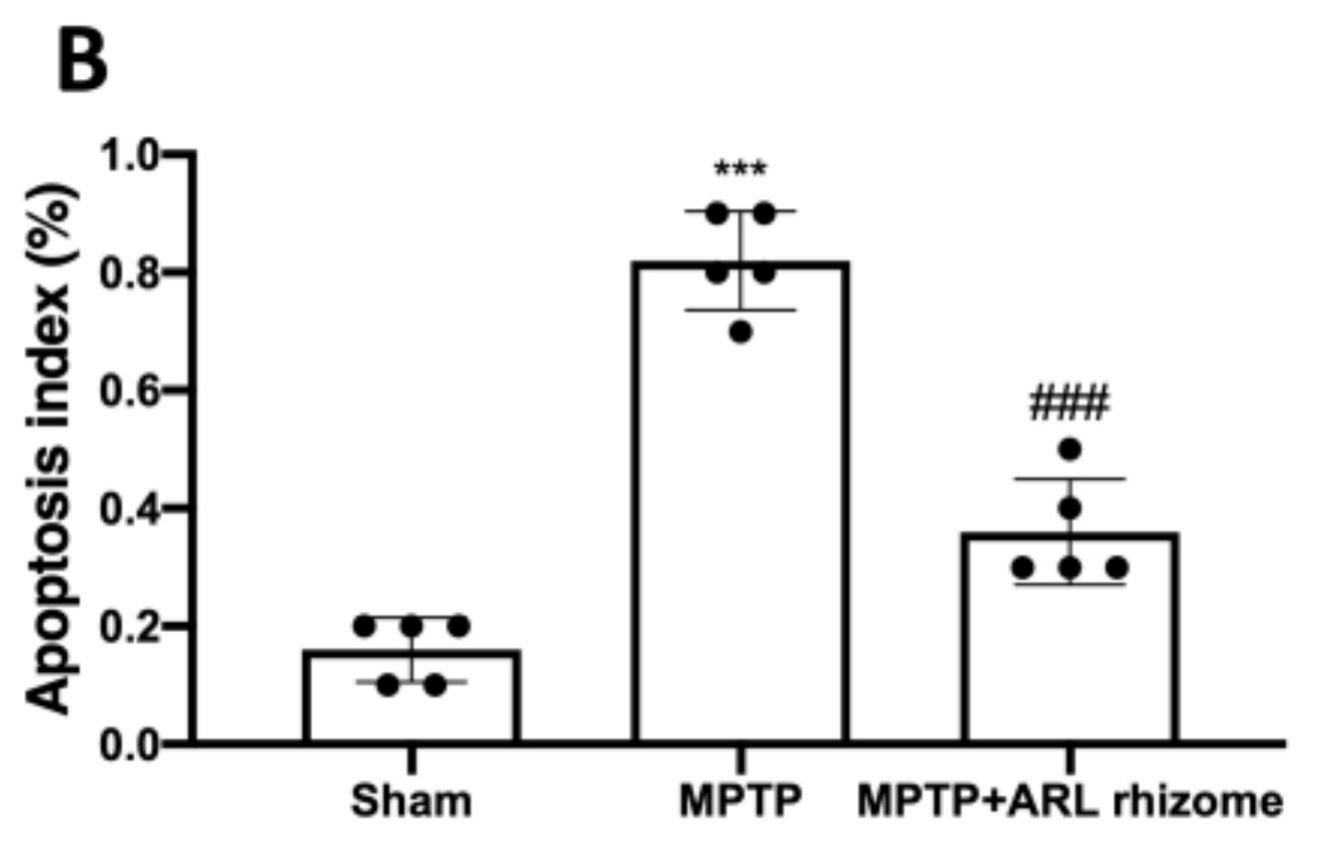
Effect of ARL rhizome on dopaminergic neurons death after MPTP-injection. Immunofluorescence for TH and TUNEL staining was evaluated in Sham; MPTP and MPTP + ARL rhizome (**A**). The results are expressed as % of apoptotic cells (**B**). The images are figurative of at least three independent experiments. Values = means ± SD of 5 animals in each group. *** *p* < 0.001 vs. Sham; ### *p* < 0.001 vs. MPTP.

We used double TH/TUNEL labeling to see if dopaminergic neurons were responsible for cell death. We detected considerable colocalization in the MPTP group compared to the Sham group by labeling dopaminergic neurons with the TH antibody, and then performed the TUNEL assay, whereas the ARL rhizome treatment considerably decreased the death of these neurons (Figure 4A,B).

### 3.4. Actaea racemosa L. Rhizome Treatment Reduced Loss of TH and DAT Expression and a-Syn Aggregation

**Figure 5:**
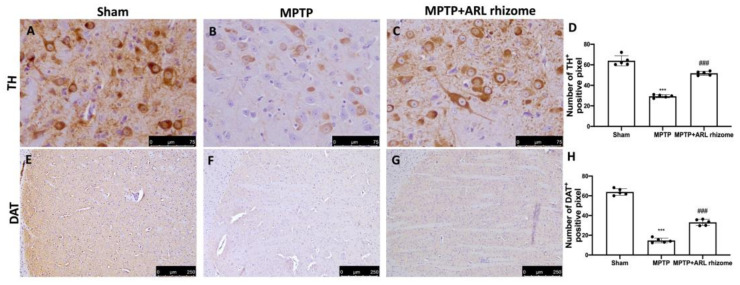
Effects of ARL rhizome on TH and DAT expression after MPTP-intoxication. Immunohistochemistry for TH was evaluated in Sham (**A**), MPTP (**B**) and MPTP + ARL rhizome (**C**). The results are expressed as number of TH^+^ positive pixel (**D**). The same analysis was performed for DAT on the brain sections of the Sham (**E**), MPTP (**F**), MPTP + ARL rhizome (**G**) groups. The results are expressed as number of DAT^+^ positive pixel (**H**). The images are figurative of at least three independent experiments. Values = means ± SD of 5 animals in each group. *** *p* < 0.001 vs. Sham; ### *p* < 0.001 vs. MPTP.

We looked at the expression of TH and DAT to see how ARL rhizome administration affected the dopamine pathway. A considerable loss of TH-positive cells was visible in the midbrain 8 days after MPTP intoxication (Figure 5B, densitometry analysis Figure 5D) compared to the Sham group (Figure 5A, densitometry analysis Figure 5D), while ARL rhizome treatment appreciably reduced the loss of TH-positive neurons in the midbrain (Figure 5C, densitometry analysis Figure 5D). We also noticed a similar pattern in DAT expression. In summary, a major decrease of DAT was detected in MPTP-injected mice (Figure 5F, densitometry analysis Figure 5H) compared to the Sham group (Figure 5E, densitometry analysis Figure 5H), but the ARL rhizome treatment significantly restored DAT levels (Figure 5G, densitometry analysis Figure 5H).

To determine the ability of the ARL rhizome to counteract the neurodegenerative process we wanted to evaluate the expression of α-syn, as the accumulation of this protein is a characteristic of PD and represents the main constituent of intraneuronal protein aggregates known as Lewy bodies.

**Figure 6:**
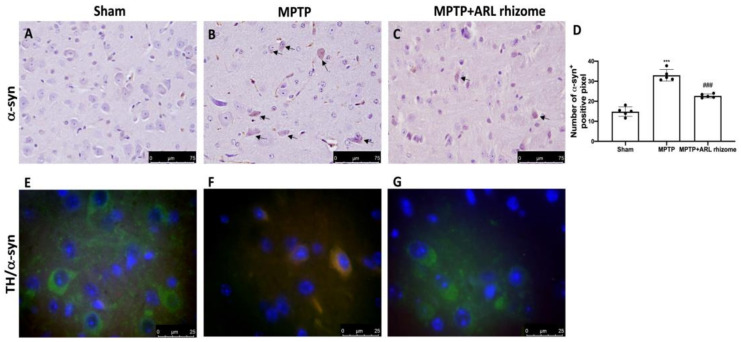
Effects of ARL rhizome on α-syn expression after MPTP-intoxication. Immunohistochemistry for α-syn was evaluated in Sham (**A**), MPTP (**B**) and MPTP + ARL rhizome (**C**). The results are expressed as number of a-syn^+^ positive pixel (**D**). The images are figurative of at least three independent experiments. Immunofluorescence for TH and a-syn was evaluated in Sham (**E**), MPTP (**F**) and MPTP + ARL rhizome (**G**). Values = means ± SD of five animals in each group. *** *p* < 0.001 vs. Sham; ### *p* < 0.001 vs. MPTP.

In comparison to the Sham group, our immunohistochemical study revealed significant immunoreactivity in MPTP-damaged mice (Figure 6B, densitometry analysis Figure 6D) (Figure 6A, densitometry analysis Figure 6D). ARL rhizome treatment, on the other hand, drastically reduced α-syn expression (Figure 6C, densitometry analysis Figure 6D).

Furthermore, to show that a-syn accumulation occurred in dopaminergic neurons, we used immunofluorescence analysis to doubly stain TH (green) and α-syn (red). We found no α-syn in TH-positive dopaminergic neurons in the Sham group (Figure 6E), but there was an increase in α-syn accumulation in TH-positive neurons following MPTP intoxication (Figure 6F). In dopaminergic neurons, ARL rhizome treatment inhibited α-syn aggregation (Figure 6G).

### 3.5. Effect of Actaea racemosa L. Rhizome Treatment on Histological Changes and Behavioral Impairments Induced by MPTP Administration

**Figure 7:**
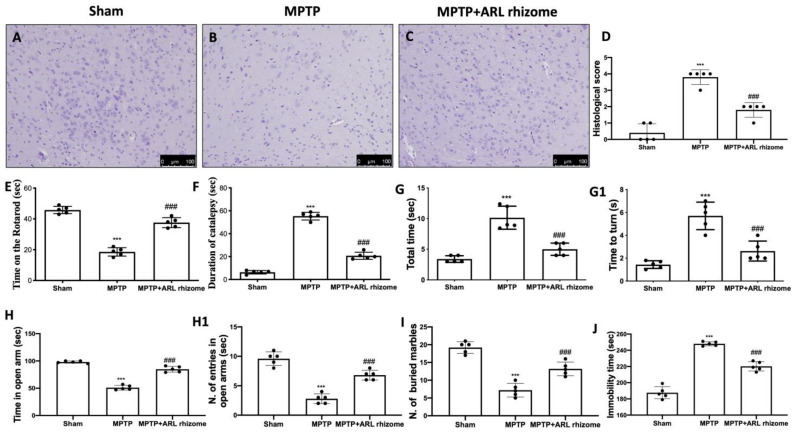
Effect of ARL rhizome on histological parameters and behavioral alterations in MPTP-lesioned mice. MPTP-injured mice were characterized by nigrostriatal dopaminergic degeneration, which translated into neuronal cell loss in the midbrain (**B**) with respect to physiological neuronal cell structure observed in control mice (**A**), ARL rhizome treatment reduced alteration of the dopaminergic tract, mitigating neuronal cell loss (**C**). See histological score (**D**). Motor function was assessed using RT (**E**), Catalepsy test (**F**) and a Pole test (**G**,**G1**). The behavioral analysis of non-motor symptoms was assessed using EPM test (**H**,**H1**), MBT (**I**) and TST (**J**). The data are representative of at least three independent experiments and are expressed as the mean ± SD of five mice for each group. *** *p* < 0.001 vs. Sham; ### *p* < 0.001 vs. MPTP.

H&E staining revealed that MPTP administration caused histological alterations in the midbrain. Sham mice had normal brain architecture and a normal number of neurons (Figure 7A,D). Instead, MPTP-treated mice displayed significant changes in normal brain architecture as well as a visible loss of neuronal cells (Figure 7B,D). In contrast, ARL rhizome-treated animals had a significant drop in the number of cells with pycnotic nuclei in the region of interest (Figure 7C,D).

The data at time 0 are not shown since no significant changes between the groups were found. Motor coordination was assessed using the RT. MPTP-treated mice demonstrated severe motor incoordination after 8 days of PD induction, as evidenced by a decrease in time spent on the Rotarod and an increase in the number of falls. In contrast, the motor impairments in the ARL rhizome-treated mice were significantly reduced (Figure 7E). MPTP also had a significant cataleptic effect in mice. In fact, 8 days after receiving MPTP, the mice displayed a considerable rise in cataleptic symptoms. Daily ARL rhizome administration, on the other hand, dramatically shortened the duration of MPTP-induced catalepsy (Figure 7F). We used the PT to assess MPTP-induced bradykinesia. When compared to the Sham group, “turn time” and “total time” increased dramatically following MPTP injection. Treatment with ARL rhizome considerably reduced “total time” and “turnaround time”, implying a considerable reduction in bradykinesia (Figure 7G,G1).

Furthermore, at the 8th day after MPTP intoxication, mice had non-motor PD signs. In the EPM, mice were observed for anxiety-like behavior. The behavioral test that we conducted revealed a significant increase in the duration spent in the open arms and the number of entries in the open arms following ARL rhizome treatment as compared to the MPTP group (Figure 7H,H1). This increase in anxiety observed in MPTP-treated mice was also confirmed by the MBT. In this test, MPTP-treated mice tended to leave significantly more glass beads unburied than mice from the Sham group. ARL rhizome treatment reduced the animals’ anxiety state, demonstrated by the number of buried marbles found, which was similar to the control group (Figure 7I).

The depression that often accompanies anxiety is another important non-motor feature of PD. TST was used to measure depression-like behavior on day 8 after MPTP intoxication. When compared to Sham mice, these mice were much more immobile during the 6 min interval. Treatment with ARL rhizome reduced immobility time to values comparable to the Sham group (Figure 7J).

## 4. Discussion

Numerous investigations have found a clear role for oxidative stress and neuroinflammation in the etiology of PD [78]. In this regard, several researchers are trying to develop anti-inflammatory and antioxidant drugs capable of counteracting these processes that play a crucial role in PD. In recent years, it has been shown that natural compounds have beneficial effects on health, as many of these products can act on aging and neurodegeneration [67,79,80,81]. Given the analgesic, antioxidant, anti-inflammatory, antipyretic and antiviral properties of *Actaea racemosa* L. [82], in the present study we wanted to evaluate its beneficial effects for the first time in an experimental model of MPTP-induced PD.

Our findings suggest that ARL rhizome can inhibit not just oxidative stress but also the inflammatory response and apoptosis, hence preventing a-syn buildup, dopaminergic neuron loss, and behavioral impairments.

Nrf2, a transcription factor that activates genes with cytoprotective functions, is involved in antioxidant and anti-inflammatory reactions [83,84]. Several phase II detoxification enzymes, including NQO1, HO-1, and many others, are encoded by these cytoprotective genes [85,86]. In an in vivo study, *Actaea racemosa* L. rhizome has already been shown to upregulate the Nrf2/HO-1 pathway [52]. Therefore, our theory is that ARL rhizome can perform its neuroprotective action through the activation of the Nrf2/HO-1/NQO1 axis. In comparison to the MPTP group, our data demonstrated that ARL rhizome dramatically increased Nrf2, HO-1, and NQO1 levels. Therefore, ARL rhizome activates the antioxidant response by restoring the redox balance.

Excessive oxidative stress induces activation of NF-kB, indicating a relationship between these two pathways [87,88]. NF-kB is a transcription factor that regulates the inflammatory response by activating several genes that code for pro-inflammatory cytokines and immunoregulatory mediators [89]. Our findings show that ARL rhizome can block NF-kB nuclear translocation and IkB-α degradation. This shows that Nrf2 regulation of redox homeostasis likely contributes to the modulation of NF-kB activity and the inflammatory response seen in PD. This pathology is also characterized by neuroinflammatory changes associated with the activation of astrocytes and microglia [50,51,90]. Several previous studies have demonstrated the phenomenon of astrogliosis and microgliosis in the MPTP-induced PD model. Our ARL rhizome treatment was able to contain the activation of GFAP and Iba-1. Once we observed the effects of ARL rhizome on oxidative stress and neuroinflammation, we moved on to the evaluation of the apoptotic process by investigating two specific markers such as Bax (pro-apoptotic protein) and Bcl-2 (anti-apoptotic protein). Our study found that by limiting the redox imbalance and the activation of inflammatory processes induced by MPTP, the ARL rhizome was able to reduce neuronal death and in particular the death of dopaminergic neurons, as demonstrated by the double staining TUNEL/TH. Degeneration is usually accompanied by a-syn buildup and a decrease in dopamine due to the death of dopaminergic neurons [91]. Given the protective role of ARL rhizome on the latter, we evaluated the ability of the ARL rhizome to reduce the loss of dopamine and the accumulation of a-synuclein by analyzing specific markers such as DAT and α-syn. The results obtained revealed that ARL rhizome is also able to act on these specific parameters of PD and on the motor and cognitive alterations that follow.

## 5. Conclusions and Limitations

In conclusion, our results demonstrated that ARL rhizome, probably acting through the Nrf2 pathway, is able to act as a potent antioxidant, reducing MPTP-induced oxidative stress, and is able to work through an indirect mechanism of acting on neuroinflammation by preventing the death of dopaminergic neurons and the aggregation of α-syn.

Obviously, the administration of ARL rhizome does not cure PD, but we can certainly say that the ARL rhizome represents a nutritional product that could limit the progression of the disease and improve the symptoms associated with this pathology.

It is obvious that the present study has limitations related to the characterization of the ARL rhizome. In fact, the goal of future studies will be to identify which components of ARL are responsible for the antioxidant and anti-inflammatory action in PD. We hypothesize that an important role may be played by hydroxycinnamic acid derivatives such as caffeic acid, ferulic and isoferulic acid, but also by triterpenes. Naturally, this hypothesis will have to be verified with specific studies both in vitro and in vivo.

## Data Availability

Based upon the rules of our laboratory, the datasets used in the current study are available from the corresponding author (dimpellizzeri@unime.it) on reasonable request.

## References

[1] Braak H., Del Tredici K., Rub U., de Vos R.A., Steur E.N.J., Braak E. (2003). Staging of brain pathology related to sporadic Parkinson’s disease. Neurobiol. Aging.

[2] Schapira A.H., Jenner P. (2011). Etiology and pathogenesis of Parkinson’s disease. Mov. Disord..

[3] Tysnes O.B., Storstein A. (2017). Epidemiology of Parkinson’s disease. J. Neural. Transm..

[4] Langston J.W., Ballard P., Tetrud J.W., Irwin I. (1983). Chronic Parkinsonism in humans due to a product of meperidine-analog synthesis. Science.

[5] Masato A., Bubacco L., Greggio E. (2021). Too much for your own good: Excessive dopamine damages neurons and contributes to Parkinson’s disease: An Editorial Highlight for “Enhanced tyrosine hydroxylase activity induces oxidative stress, causes accumulation of autotoxic catecholamine metabolites, and augments amphetamine effects in vivo”. J. Neurochem..

[6] Tambasco N., Romoli M., Calabresi P. (2018). Levodopa in Parkinson’s Disease: Current Status and Future Developments. Curr. Neuropharmacol..

[7] Marino B.L.B., de Souza L.R., Sousa K.P.A., Ferreira J.V., Padilha E.C., da Silva C., Taft C.A., Hage-Melim L.I.S. (2020). Parkinson’s Disease: A Review from Pathophysiology to Treatment. Mini Rev. Med. Chem..

[8] Corona J.C. (2018). Natural Compounds for the Management of Parkinson’s Disease and Attention-Deficit/Hyperactivity Disorder. BioMed Res. Int..

[9] Rahman M.H., Bajgai J., Fadriquela A., Sharma S., Trinh T.T., Akter R., Jeong Y.J., Goh S.H., Kim C.S., Lee K.J. (2021). Therapeutic Potential of Natural Products in Treating Neurodegenerative Disorders and Their Future Prospects and Challenges. Molecules.

[10] D’Amico R., Cordaro M., Siracusa R., Impellizzeri D., Trovato Salinaro A., Scuto M., Ontario M.L., Crea R., Cuzzocrea S., Di Paola R. (2021). Wnt/beta-Catenin Pathway in Experimental Model of Fibromyalgia: Role of Hidrox((R)). Biomedicines.

[11] D’Amico R., Trovato Salinaro A., Cordaro M., Fusco R., Impellizzeri D., Interdonato L., Scuto M., Ontario M.L., Crea R., Siracusa R. (2021). Hidrox((R)) and Chronic Cystitis: Biochemical Evaluation of Inflammation, Oxidative Stress, and Pain. Antioxidants.

[12] Siracusa R., Monaco F., D’Amico R., Genovese T., Cordaro M., Interdonato L., Gugliandolo E., Peritore A.F., Crupi R., Cuzzocrea S. (2021). Epigallocatechin-3-Gallate Modulates Postoperative Pain by Regulating Biochemical and Molecular Pathways. Int. J. Mol. Sci..

[13] Siracusa R., Fusco R., Peritore A.F., Cordaro M., D’Amico R., Genovese T., Gugliandolo E., Crupi R., Smeriglio A., Mandalari G. (2020). The Antioxidant and Anti-Inflammatory Properties of Anacardium occidentale L. Cashew Nuts in a Mouse Model of Colitis. Nutrients.

[14] Houghton P.J., Howes M.J. (2005). Natural products and derivatives affecting neurotransmission relevant to Alzheimer’s and Parkinson’s disease. Neurosignals.

[15] Sengupta T., Vinayagam J., Singh R., Jaisankar P., Mohanakumar K.P. (2016). Plant-Derived Natural Products for Parkinson’s Disease Therapy. Adv. Neurobiol..

[16] Cilia R., Laguna J., Cassani E., Cereda E., Pozzi N.G., Isaias I.U., Contin M., Barichella M., Pezzoli G. (2017). Mucuna pruriens in Parkinson disease: A double-blind, randomized, controlled, crossover study. Neurology.

[17] Sengupta T., Vinayagam J., Nagashayana N., Gowda B., Jaisankar P., Mohanakumar K.P. (2011). Antiparkinsonian effects of aqueous methanolic extract of Hyoscyamus niger seeds result from its monoamine oxidase inhibitory and hydroxyl radical scavenging potency. Neurochem. Res..

[18] Ahmad M., Saleem S., Ahmad A.S., Ansari M.A., Yousuf S., Hoda M.N., Islam F. (2005). Neuroprotective effects of Withania somnifera on 6-hydroxydopamine induced Parkinsonism in rats. Hum. Exp. Toxicol..

[19] Manjunath M.J., Muralidhara (2015). Standardized extract of Withania somnifera (Ashwagandha) markedly offsets rotenone-induced locomotor deficits, oxidative impairments and neurotoxicity in Drosophila melanogaster. J. Food Sci. Technol..

[20] Prakash J., Chouhan S., Yadav S.K., Westfall S., Rai S.N., Singh S.P. (2014). Withania somnifera alleviates parkinsonian phenotypes by inhibiting apoptotic pathways in dopaminergic neurons. Neurochem. Res..

[21] Rojas P., Ruiz-Sanchez E., Rojas C., Ogren S.O. (2012). Ginkgo biloba extract (EGb 761) modulates the expression of dopamine-related genes in 1-methyl-4-phenyl-1,2,3,6-tetrahydropyridine-induced Parkinsonism in mice. Neuroscience.

[22] Yu D., Zhang P., Li J., Liu T., Zhang Y., Wang Q., Zhang J., Lu X., Fan X. (2021). Neuroprotective effects of Ginkgo biloba dropping pills in Parkinson’s disease. J. Pharm. Anal..

[23] Ma Y., Cong W., Huang H., Sun L., Mai A.H., Boonen K., Maryam W., De Borggraeve W., Luo G., Liu Q. (2019). Identification of fukinolic acid from Cimicifuga heracleifolia and its derivatives as novel antiviral compounds against enterovirus A71 infection. Int. J. Antimicrob. Agents.

[24] Yun J.W., You J.R., Kim Y.S., Cho E.Y., Kim S.H., Yoon J.H., Kwon E., Chung D.H., Kim Y.T., Jang J.J. (2015). Pre-clinical in vitro and in vivo safety evaluation of Cimicifuga heracleifolia. Regul. Toxicol. Pharmacol..

[25] Mahady G.B., Fong H.H., Farnsworth N.R. (2001). Botanical Dietary Supplements.

[26] Barrett M.L. (2004). Handbook of Clinically Tested Herbal Remedies.

[27] Borrelli F., Ernst E. (2002). *Cimicifuga racemosa*: A systematic review of its clinical efficacy. Eur. J. Clin. Pharmacol..

[28] Kronenberg F., Fugh-Berman A. (2002). Complementary and alternative medicine for menopausal symptoms: A review of randomized, controlled trials. Ann. Intern. Med..

[29] D’Amicon R., Impellizzeri D., Cuzzocrea S., Di Paola R. (2020). Aliamides update: Palmitoylethanolamide and its formulations on management of peripheral neuropathic pain. Int. J. Mol. Sci..

[30] Wuttke W., Seidlova-Wuttke D., Gorkow C. (2003). The Cimicifuga preparation BNO 1055 vs. conjugated estrogens in a double-blind placebo-controlled study: Effects on menopause symptoms and bone markers. Maturitas.

[31] Borrelli F., Izzo A., Ernst E. (2003). Pharmacological effects of *Cimicifuga racemosa*. Life Sci..

[32] Burdette J.E., Liu J., Chen S.-N., Fabricant D.S., Piersen C.E., Barker E.L., Pezzuto J.M., Mesecar A., Van Breemen R.B., Farnsworth N.R. (2003). Black cohosh acts as a mixed competitive ligand and partial agonist of the serotonin receptor. J. Agric. Food Chem..

[33] Fabricant D.S., Nikolic D., Lankin D.C., Chen S.-N., Jaki B.U., Krunic A., van Breemen R.B., Fong H.H., Farnsworth N.R., Pauli G.F. (2005). Cimipronidine, a Cyclic Guanidine Alkaloid from Cimicifuga r acemosa. J. Nat. Prod..

[34] He K., Pauli G.F., Zheng B., Wang H., Bai N., Peng T., Roller M., Zheng Q. (2006). Cimicifuga species identification by high performance liquid chromatography-photodiode array/mass spectrometric/evaporative light scattering detection for quality control of black cohosh products. J. Chromatogr. A.

[35] Mohapatra S., Iqubal A., Ansari M.J., Jan B., Zahiruddin S., Mirza M.A., Ahmad S., Iqbal Z. (2022). Benefits of Black Cohosh (*Cimicifuga racemosa*) for Women Health: An Up-Close and In-Depth Review. Pharmaceuticals.

[36] Lim J.O., Song K.H., Lee I.S., Lee S.J., Kim W.I., Pak S.W., Shin I.S., Kim T. (2021). Cimicifugae Rhizoma Extract Attenuates Oxidative Stress and Airway Inflammation via the Upregulation of Nrf2/HO-1/NQO1 and Downregulation of NF-kappa B Phosphorylation in Ovalbumin-Induced Asthma. Antioxidants.

[37] Kruse S.O., Lohning A., Pauli G.F., Winterhoff H., Nahrstedt A. (1999). Fukiic and piscidic acid esters from the rhizome of *Cimicifuga racemosa* and the in vitro estrogenic activity of fukinolic acid. Planta Med..

[38] Li W.K., Chen S.N., Fabricant D., Angerhofer C.K., Fong H.H.S., Farnsworth N.R., Fitzloff J.F. (2002). High-performance liquid chromatographic analysis of Black Cohosh (*Cimicifuga racemosa*) constituents with in-line evaporative light scattering and photodiode array detection. Anal. Chim. Acta.

[39] Liby K.T., Yore M.M., Sporn M.B. (2007). Triterpenoids and rexinoids as multifunctional agents for the prevention and treatment of cancer. Nat. Rev. Cancer.

[40] Sahreen S., Khan M.R., Khan R.A. (2011). Hepatoprotective effects of methanol extract of Carissa opaca leaves on CCl4-induced damage in rat. BMC Complement. Altern. Med..

[41] Burdette J.E., Chen S.N., Lu Z.Z., Xu H.Y., White B.E.P., Fabricant D.S., Liu J.H., Fong H.H.S., Farnsworth N.R., Constantinou A.I. (2002). Black cohosh (*Cimicifuga racemosa* L.) protects against menadione-induced DNA damage through scavenging of reactive oxygen species: Bioassay-directed isolation and characterization of active principles. J. Agric. Food Chem..

[42] Liao X., Zhang Q.Y., Xu L., Zhang H.Y. (2020). Potential Targets of Actein Identified by Systems Chemical Biology Methods. ChemMedChem.

[43] Cicek S.S., Girreser U., Zidorn C. (2018). Quantification of the total amount of black cohosh cycloartanoids by integration of one specific H-1 NMR signal. J. Pharm. Biomed..

[44] Li H.X., Yu Z.Y. (2006). Cimicifugae rhizoma: From origins, bioactive constituents to clinical outcomes. Curr. Med. Chem..

[45] Avula B., Wang Y.H., Smillie T.J., Khan I.A. (2009). Quantitative Determination of Triterpenoids and Formononetin in Rhizomes of Black Cohosh (Actaea racemosa) and Dietary Supplements by Using UPLC-UV/ELS Detection and Identification by UPLC-MS. Planta Med..

[46] Nikolic D., Lankin D.C., Cisowska T., Chen S.N., Pauli G.F., van Breemen R.B. (2015). Nitrogen-Containing Constituents of Black Cohosh: Chemistry, Structure Elucidation, and Biological Activities. Recent Adv. Phytochem..

[47] Li X., Lin J., Gao Y., Han W., Chen D. (2012). Antioxidant activity and mechanism of Rhizoma Cimicifugae. Chem. Cent. J..

[48] Bae N., Ahn T., Chung S., Oh M.S., Ko H., Oh H., Park G., Yang H.O. (2011). The neuroprotective effect of modified Yeoldahanso-tang via autophagy enhancement in models of Parkinson’s disease. J. Ethnopharmacol..

[49] Keshavarzi Z., Shakeri F., Barreto G.E., Bibak B., Sathyapalan T., Sahebkar A. (2019). Medicinal plants in traumatic brain injury: Neuroprotective mechanisms revisited. Biofactors.

[50] Siracusa R., Paterniti I., Cordaro M., Crupi R., Bruschetta G., Campolo M., Cuzzocrea S., Esposito E. (2018). Neuroprotective Effects of Temsirolimus in Animal Models of Parkinson’s Disease. Mol. Neurobiol..

[51] Cordaro M., Siracusa R., Crupi R., Impellizzeri D., Peritore A.F., D’Amico R., Gugliandolo E., Di Paola R., Cuzzocrea S. (2018). 2-Pentadecyl-2-Oxazoline Reduces Neuroinflammatory Environment in the MPTP Model of Parkinson Disease. Mol. Neurobiol..

[52] Peritore A.F., Crupi R., Scuto M., Gugliandolo E., Siracusa R., Impellizzeri D., Cordaro M., D’Amico R., Fusco R., Di Paola R. (2020). The role of annexin A1 and formyl peptide receptor 2/3 signaling in chronic corticosterone-induced depression-like behaviors and impairment in hippocampal-dependent memory. CNS Neurol. Disord.-Drug Targets.

[53] Paterniti I., Campolo M., Siracusa R., Cordaro M., Di Paola R., Calabrese V., Navarra M., Cuzzocrea S., Esposito E. (2017). Liver X receptors activation, through TO901317 binding, reduces neuroinflammation in Parkinson’s disease. PLoS ONE.

[54] Mariotto S., Esposito E., Di Paola R., Ciampa A., Mazzon E., de Prati A.C., Darra E., Vincenzi S., Cucinotta G., Caminiti R. (2008). Protective effect of Arbutus unedo aqueous extract in carrageenan-induced lung inflammation in mice. Pharm. Res..

[55] Di Paola D., Iaria C., Capparucci F., Cordaro M., Crupi R., Siracusa R., D’Amico R., Fusco R., Impellizzeri D., Cuzzocrea S. (2021). Aflatoxin B1 Toxicity in Zebrafish Larva (Danio rerio): Protective Role of Hericium erinaceus. Toxins.

[56] Petrosino S., Campolo M., Impellizzeri D., Paterniti I., Allara M., Gugliandolo E., D’Amico R., Siracusa R., Cordaro M., Esposito E. (2017). 2-Pentadecyl-2-Oxazoline, the Oxazoline of Pea, Modulates Carrageenan-Induced Acute Inflammation. Front. Pharm..

[57] Impellizzeri D., Siracusa R., Cordaro M., Crupi R., Peritore A.F., Gugliandolo E., D’Amico R., Petrosino S., Evangelista M., Di Paola R. (2019). N-Palmitoylethanolamine-oxazoline (PEA-OXA): A new therapeutic strategy to reduce neuroinflammation, oxidative stress associated to vascular dementia in an experimental model of repeated bilateral common carotid arteries occlusion. Neurobiol. Dis..

[58] Mazzon E., Esposito E., Impellizzeri D., Di Paola R., Melani A., Bramanti P., Pedata F., Cuzzocrea S. (2011). CGS 21680, an agonist of the adenosine (A2A) receptor, reduces progression of murine type II collagen-induced arthritis. J. Rheumatol..

[59] Fusco R., Cordaro M., Siracusa R., D’Amico R., Genovese T., Gugliandolo E., Peritore A.F., Crupi R., Impellizzeri D., Cuzzocrea S. (2020). Biochemical Evaluation of the Antioxidant Effects of Hydroxytyrosol on Pancreatitis-Associated Gut Injury. Antioxidants.

[60] Cuzzocrea S., Mazzon E., Paola R.D., Genovese T., Muia C., Caputi A.P., Salvemini D. (2005). Effects of combination M40403 and dexamethasone therapy on joint disease in a rat model of collagen-induced arthritis. Arthritis Rheum..

[61] Siracusa R., Paterniti I., Bruschetta G., Cordaro M., Impellizzeri D., Crupi R., Cuzzocrea S., Esposito E. (2016). The Association of Palmitoylethanolamide with Luteolin Decreases Autophagy in Spinal Cord Injury. Mol. Neurobiol..

[62] Lunardelli M.L., Crupi R., Siracusa R., Cocuzza G., Cordaro M., Martini E., Impellizzeri D., Di Paola R., Cuzzocrea S. (2019). Co-ultraPEALut: Role in Preclinical and Clinical Delirium Manifestations. CNS Neurol. Disord. Drug Targets.

[63] Impellizzeri D., Siracusa R., Cordaro M., Peritore A.F., Gugliandolo E., Mancuso G., Midiri A., Di Paola R., Cuzzocrea S. (2018). Therapeutic potential of dinitrobenzene sulfonic acid (DNBS)-induced colitis in mice by targeting IL-1beta and IL-18. Biochem. Pharm..

[64] Gugliandolo E., Peritore A.F., D’Amico R., Licata P., Crupi R. (2020). Evaluation of Neuroprotective Effects of Quercetin against Aflatoxin B1-Intoxicated Mice. Animals.

[65] Siracusa R., Paterniti I., Impellizzeri D., Cordaro M., Crupi R., Navarra M., Cuzzocrea S., Esposito E. (2015). The Association of Palmitoylethanolamide with Luteolin Decreases Neuroinflammation and Stimulates Autophagy in Parkinson’s Disease Model. CNS Neurol. Disord. Drug Targets.

[66] Cordaro M., Siracusa R., Impellizzeri D., D’Amico R., Peritore A.F., Crupi R., Gugliandolo E., Fusco R., Di Paola R., Schievano C. (2019). Safety and efficacy of a new micronized formulation of the ALIAmide palmitoylglucosamine in preclinical models of inflammation and osteoarthritis pain. Arthritis Res..

[67] D’Amico R., Trovato Salinaro A., Fusco R., Cordaro M., Impellizzeri D., Scuto M., Ontario M.L., Lo Dico G., Cuzzocrea S., Di Paola R. (2021). Hericium erinaceus and Coriolus versicolor Modulate Molecular and Biochemical Changes after Traumatic Brain Injury. Antioxidants.

[68] Son Y., Yang M., Kang S., Lee S., Kim J., Kim J., Park S., Kim J.S., Jo S.K., Jung U. (2015). Cranial irradiation regulates CREB-BDNF signaling and variant BDNF transcript levels in the mouse hippocampus. Neurobiol. Learn. Mem..

[69] Fleming S.M., Mulligan C.K., Richter F., Mortazavi F., Lemesre V., Frias C., Zhu C., Stewart A., Gozes I., Morimoto B. (2011). A pilot trial of the microtubule-interacting peptide (NAP) in mice overexpressing alpha-synuclein shows improvement in motor function and reduction of alpha-synuclein inclusions. Mol. Cell Neurosci..

[70] Araki T., Kumagai T., Tanaka K., Matsubara M., Kato H., Itoyama Y., Imai Y. (2001). Neuroprotective effect of riluzole in MPTP-treated mice. Brain Res..

[71] Genovese T., Siracusa R., Fusco R., D’Amico R., Impellizzeri D., Peritore A.F., Crupi R., Gugliandolo E., Morabito R., Cuzzocrea S. (2021). Atrazine Inhalation Causes Neuroinflammation, Apoptosis and Accelerating Brain Aging. Int. J. Mol. Sci..

[72] Sedelis M., Schwarting R.K., Huston J.P. (2001). Behavioral phenotyping of the MPTP mouse model of Parkinson’s disease. Behav. Brain Res..

[73] Pellow S., Chopin P., File S.E., Briley M. (1985). Validation of open: Closed arm entries in an elevated plus-maze as a measure of anxiety in the rat. J. Neurosci. Methods.

[74] Cordaro M., Salinaro A.T., Siracusa R., D’Amico R., Impellizzeri D., Scuto M., Ontario M.L., Cuzzocrea S., Di Paola R., Fusco R. (2021). Key Mechanisms and Potential Implications of Hericium erinaceus in NLRP3 Inflammasome Activation by Reactive Oxygen Species during Alzheimer’s Disease. Antioxidants.

[75] Wada M., Ang M.J., Weerasinghe-Mudiyanselage P.D.E., Kim S.H., Kim J.C., Shin T., Moon C. (2021). Behavioral characterization in MPTP/p mouse model of Parkinson’s disease. J. Integr. Neurosci..

[76] Steru L., Chermat R., Thierry B., Simon P. (1985). The tail suspension test: A new method for screening antidepressants in mice. Psychopharmacology.

[77] Di Paola R., Impellizzeri D., Fusco R., Cordaro M., Siracusa R., Crupi R., Esposito E., Cuzzocrea S. (2016). Ultramicronized palmitoylethanolamide (PEA-um((R))) in the treatment of idiopathic pulmonary fibrosis. Pharm. Res..

[78] Jayaram S., Krishnamurthy P.T. (2021). Role of microgliosis, oxidative stress and associated neuroinflammation in the pathogenesis of Parkinson’s disease: The therapeutic role of Nrf2 activators. Neurochem. Int..

[79] Carrera I., Cacabelos R. (2019). Current Drugs and Potential Future Neuroprotective Compounds for Parkinson’s Disease. Curr. Neuropharmacol..

[80] Crupi R., Impellizzeri D., Cordaro M., Siracusa R., Casili G., Evangelista M., Cuzzocrea S. (2018). N-palmitoylethanolamide Prevents Parkinsonian Phenotypes in Aged Mice. Mol. Neurobiol..

[81] Siracusa R., Scuto M., Fusco R., Trovato A., Ontario M.L., Crea R., Di Paola R., Cuzzocrea S., Calabrese V. (2020). Anti-inflammatory and Anti-oxidant Activity of Hidrox((R)) in Rotenone-Induced Parkinson’s Disease in Mice. Antioxidants.

[82] Jeong S.H., Lee J.E., Kim B.B., Ko Y., Park J.B. (2015). Evaluation of the effects of Cimicifugae Rhizoma on the morphology and viability of mesenchymal stem cells. Exp. Med..

[83] Calkins M.J., Johnson D.A., Townsend J.A., Vargas M.R., Dowell J.A., Williamson T.P., Kraft A.D., Lee J.M., Li J., Johnson J.A. (2009). The Nrf2/ARE pathway as a potential therapeutic target in neurodegenerative disease. Antioxid. Redox Signal..

[84] Lastres-Becker I., Garcia-Yague A.J., Scannevin R.H., Casarejos M.J., Kugler S., Rabano A., Cuadrado A. (2016). Repurposing the NRF2 Activator Dimethyl Fumarate as Therapy Against Synucleinopathy in Parkinson’s Disease. Antioxid. Redox Signal..

[85] Calabrese V., Cornelius C., Dinkova-Kostova A.T., Calabrese E.J., Mattson M.P. (2010). Cellular stress responses, the hormesis paradigm, and vitagenes: Novel targets for therapeutic intervention in neurodegenerative disorders. Antioxid. Redox Signal..

[86] Mutter F.E., Park B.K., Copple I.M. (2015). Value of monitoring Nrf2 activity for the detection of chemical and oxidative stress. Biochem. Soc. Trans..

[87] Lingappan K. (2018). NF-kappaB in Oxidative Stress. Curr. Opin. Toxicol..

[88] Esposito E., Impellizzeri D., Bruschetta G., Cordaro M., Siracusa R., Gugliandolo E., Crupi R., Cuzzocrea S. (2016). A new co-micronized composite containing palmitoylethanolamide and polydatin shows superior oral efficacy compared to their association in a rat paw model of carrageenan-induced inflammation. Eur. J. Pharm..

[89] Barnabei L., Laplantine E., Mbongo W., Rieux-Laucat F., Weil R. (2021). NF-kappaB: At the Borders of Autoimmunity and Inflammation. Front. Immunol..

[90] Flores-Cuadrado A., Saiz-Sanchez D., Mohedano-Moriano A., Lamas-Cenjor E., Leon-Olmo V., Martinez-Marcos A., Ubeda-Banon I. (2021). Astrogliosis and sexually dimorphic neurodegeneration and microgliosis in the olfactory bulb in Parkinson’s disease. NPJ Park. Dis..

[91] Gomez-Benito M., Granado N., Garcia-Sanz P., Michel A., Dumoulin M., Moratalla R. (2020). Modeling Parkinson’s Disease with the Alpha-Synuclein Protein. Front. Pharm..

